# Cytosolic 5′-Nucleotidase II Is a Sensor of Energy Charge and Oxidative Stress: A Possible Function as Metabolic Regulator

**DOI:** 10.3390/cells10010182

**Published:** 2021-01-18

**Authors:** Rossana Pesi, Simone Allegrini, Francesco Balestri, Mercedes Garcia-Gil, Federico Cividini, Laura Colombaioni, Lars Petter Jordheim, Marcella Camici, Maria Grazia Tozzi

**Affiliations:** 1 Unità di Biochimica, Dipartimento di Biologia, Università di Pisa, Via San Zeno 51, 56127 Pisa, Italy; rossana.pesi@unipi.it (R.P.); francesco.balestri@unipi.it (F.B.); marcella.camici@unipi.it (M.C.); maria.grazia.tozzi@unipi.it (M.G.T.); 2 Unità di Fisiologia Generale, Dipartimento di Biologia, Università di Pisa, Via San Zeno 31, 56127 Pisa, Italy; mercedes.garcia@unipi.it; 3 Department of Medicine, University of California, La Jolla, San Diego, CA 92093-0671, USA; fcividini@ucsd.edu; 4 Istituto di Neuroscienze, CNR, Via Giuseppe Moruzzi 1, 56124 Pisa, Italy; laura.colombaioni@in.cnr.it; 5 Centre de Recherche en Cancérologie de Lyon, INSERM U1052-CNRS UMR 5286, Faculté Rockefeller, 8 Avenue Rockefeller, 69008 Lyon, France; lars-petter.jordheim@univ-lyon1.fr

**Keywords:** Cytosolic 5′-nucleotidase II, NT5C2, energy charge, oxidative stress, AMPK, ADF, A549, IPAF

## Abstract

Cytosolic 5′-nucleotidase II (NT5C2) is a highly regulated enzyme involved in the maintenance of intracellular purine and the pyrimidine compound pool. It dephosphorylates mainly IMP and GMP but is also active on AMP. This enzyme is highly expressed in tumors, and its activity correlates with a high rate of proliferation. In this paper, we show that the recombinant purified NT5C2, in the presence of a physiological concentration of the inhibitor inorganic phosphate, is very sensitive to changes in the adenylate energy charge, especially from 0.4 to 0.9. The enzyme appears to be very sensitive to pro-oxidant conditions; in this regard, the possible involvement of a disulphide bridge (C175-C547) was investigated by using a C547A mutant NT5C2. Two cultured cell models were used to further assess the sensitivity of the enzyme to oxidative stress conditions. NT5C2, differently from other enzyme activities, was inactivated and not rescued by dithiothreitol in a astrocytoma cell line (ADF) incubated with hydrogen peroxide. The incubation of a human lung carcinoma cell line (A549) with 2-deoxyglucose lowered the cell energy charge and impaired the interaction of NT5C2 with the ice protease-activating factor (IPAF), a protein involved in innate immunity and inflammation.

## 1. Introduction

NT5C2 is a highly regulated enzyme widely expressed in vertebrates involved in the catabolism of purine nucleoside monophosphates. The enzyme preferentially hydrolyzes IMP but, also, GMP, UMP and AMP [1], and, by virtue of its catalytic mechanism, NT5C2 is also able to transfer a phosphate group from a nucleoside monophosphate donor to a nucleoside acceptor (phosphotransferase activity) [2]. In the absence of positive effectors, the enzyme is almost inactive, while its activity increases approximately 20-fold in the presence of physiological concentrations of ATP and, to a minor extent, of ADP. Other phosphorylated compounds can activate NT5C2, including 2,3-bisphosphoglycerate (BPG), and diadenosine polyphosphate, mainly Ap_4_A [1,3]. A crystallographic analysis demonstrated that the enzyme displays at least two different conformations depending on the binding of the allosteric regulators [4]. A comparison of the basal (apo) and active (allosteric effector bound) structures of NT5C2 revealed a role of the G355-E364 region in the allosteric activation of the enzyme. This segment (helix A) is disordered in the apo inactive form of the enzyme and adopts an ordered α-helix conformation in the effector-bound activated state. This conformational change facilitates substrate binding and catalysis by inducing a rotation of F354 out of the active site and by moving D356 into the catalytic center [5]. The relevance of helix A in the activation of NT5C2 is supported by modeling studies implicating local conformational changes in this region as responsible for the increased nucleotidase activity of a strongly activating NT5C2 mutation (K359Q) [6]. The enzyme possesses eight cysteine residues, and two of them (C175 and C547) may form a disulphide bridge in oxidizing conditions, which determines a loss of enzyme activity that can be reverted by incubation with dithiothreitol (DTT) [7]. Therefore, this enzyme can exist in different conformations with different activities, depending upon the oxidative stress and ATP concentration.

The enzyme has been silenced or overexpressed in several cell models [1]. Some of the effects exerted by these modifications are cell-specific, but there are several interesting consequences in common among the utilized cell models. The intracellular purine and pyrimidine nucleotides are increased or unaffected by silencing, while they are decreased in overexpressing cells [8,9,10,11,12,13,14,15,16]. Similar effects were observed in cells expressing mutant forms of NT5C2 [17,18]. In several cell models, an increase of intracellular AMP was observed following NT5C2 silencing, and in some cases, a consequent activation of AMPK was indicated as responsible for some of the consequences of the low NT5C2 expression [19]. In a previous paper, we noticed a decrease of cell proliferation and motility, an increase of oxidative metabolism and a strong decrease of the rate of protein synthesis in cancer cell models after silencing NT5C2 to approximately 50% [8]. Additionally, *NT5C2*-deficient animal models were prepared to better investigate the relationship between NT5C2 activity and AMPK activation; nevertheless, the results were somehow contradictory. In fact, in *NT5C2*-deficient mice, no significant increase of AMP or AMPK activation, with respect to wild-type mice, could be demonstrated in contracting skeletal muscle [14]. This result is in contrast with previous results obtained in isolated skeletal muscle and myotubes silenced for NT5C2, although with different techniques [20]. However, in *NT5C2*-deficient mice, a tendency to reduce the body weight gain and insulin resistance induced by a high-fat diet was observed, and in this case, an involvement of AMP accumulation and AMPK activation at least in white adipose tissue could not be ruled out [15].

NT5C2 is a protein remarkably conserved among eukaryotes [1]. Indeed, *Drosophila melanogaster* possesses a protein with 80% sequence similarity with human NT5C2, indicating that the two proteins play a similar role [21]. The silencing of NT5C2 homologous in *Drosophila* causes a very significant impairment in climbing ability, indicating an involvement of the protein in the regulation of energy-consuming tasks such as motility [21]. Finally, a low expression of the *NT5C2* coding gene has been associated with disorders characterized by psychiatric and psychomotor disturbances, including schizophrenia, Parkinson’s disease and spastic paraplegia [22], leading to the hypothesis that a chronic activation of AMPK in neuronal cells could be at the basis of the mentioned syndromes [21]. In fact, the activation of AMPK and a consequent decrease of the rate of protein synthesis could be demonstrated in human neural progenitor cells knocked down for NT5C2 [21]. Since NT5C2 activity is enhanced by a high ATP concentration, it is reasonable to expect that, in well-nourished cells, a high NT5C2 activity and high AMP deaminase activity (which is also activated by ATP) contribute to regulating the internal purine nucleotides and 5-phosphoribosyl-1-pyrophosphate pools producing mainly inosine and hypoxanthine [23]. On the contrary, a decrease of ATP concentration causes a decrease of NT5C2 activity and, in turn, accumulation of AMP and IMP, potentially causing an activation of AMPK and excretion of adenosine [19]. Indeed, NT5C2 silencing, simulating a decrease of enzyme activity, might cause some of the consequences described above through AMP accumulation and adenosine production. Nevertheless, while some consequences of the alteration of NT5C2 expression were demonstrated in several cell and animal models, the expected modification of the intracellular nucleotide pools was clearly demonstrated only in a few cases [8,9,10,11,15]. Therefore, we suspect that the regulatory function of NT5C2 can be exerted also through different mechanisms. A few years ago, we demonstrated that NT5C2 can interact with ice protease-activating factor (IPAF), a protein that belongs to the family of NBS-LRR (nucleotide-binding site and leucine-rich repeat), involved in innate immunity and inflammation [24]. Since then, using different techniques, more than 40 different proteins that can interact with NT5C2 were suggested and indicated in the databases (BioGRID and IntAct-EMBL-EBI). On this basis, we hypothesized that NT5C2, as a function of energy charge and intracellular oxidative state, changes the activity and, therefore, conformation, and these modifications reflect on different regulatory systems and convey information about the health state of the cell, contributing to the cell metabolic adjustments. In this paper, we show that the conformational change of NT5C2 induced by an alteration of energy charge or of oxidative stress is associated to a different ability of the protein to bind and, possibly, to regulate its target protein(s).

## 2. Materials and Methods

### 2.1. Materials

[8-^14^C]-IMP and [8-^14^C]-inosine were purchased from Moravek Biochemicals and Radiochemicals (Brea, CA, USA); DTT, ATP, ADP, AMP, MgCl_2_ 5,5′-dithiobis(2-nitrobenzoic acid) (DTNB), 3,3′,5,5′-tetramethylbenzidine (TMB), STOP solution, primary antibody against NT5C2 and 2-deoxyglucose (2-dG) were from Sigma (Milano, Italy) and polyethyleneimine (PEI)-cellulose precoated thin-layer plastic sheets were obtained from Merck (Darmstadt, Germany) and prewashed once with 10% NaCl and three times with deionized water before use. DE-81 chromatographic paper was from Whatman (Madstone, UK), horse radish peroxidase (HRP)-linked anti-rabbit secondary antibodies were from Cell Signaling (Danvers, MA, USA) and scintillation liquid Optiphase Hisafe 2 was from Beckman (Brea, CA, USA). Acrylamide and TEMED were from Bio-Rad (Segrate, Milan); penicillin/streptomycin; glutamine and fetal bovine serum (FBS), RPMI and DMEM media were from Euroclone (Pero, Milan) and a Duolink kit for the proximity ligation assay (PLA) assay was from Olink Bioscience (St. Louis, MO, USA). The human lung carcinoma (A549) and the human astrocytoma (ADF) cell lines were purchased from ATCC (ATCC^®^ CCL-185TM) and routinely tested for *Mycoplasma* contamination by PCR. All other reagents were of reagent grade.

### 2.2. Purification of Recombinant NT5C2

The wild-type (WT) bovine recombinant NT5C2 was prepared as previously described [25]. Mutant C547A was produced as described in the QuiKChange site-directed mutagenesis kit manual (Stratagene, La Jolla, CA, USA) using the following primers: primer forward (NT5C2-C547A-F): 5’-cccccaggaaattacacacgcccatgacgaagatgatgat-3’ and primer reverse (NT5C2-C547A-R): 5’-atcatcatcttcgtcatgggcgtgtgtaatttcctggggg-3’. The purification of the recombinant enzymes was performed as described by Allegrini et al. [25]. The recombinant enzymes cannot be frozen and were stored at 4 °C in the presence of 1-M ammonium sulphate. The C547A mutant is less stable than the WT enzyme.

### 2.3. NT5C2 Activity Assays

NT5C2 was assayed both for hydrolase and phosphotransferase activity at 37 °C. The hydrolase activity was measured either with a spectrophotometric or a radioenzymatic assay. NT5C2 activity was detected by a spectrophotometric assay by measuring the increase in absorbance at 293 nm that accompanies the conversion of IMP into uric acid in the presence of an excess of purine nucleoside phosphorylase (PNP) and xanthine oxidase (XOD). The reactions were contained in a final volume of 1-mL, 2-mM IMP, 20-mM MgCl_2_, 5-mM BPG, 3-mM inorganic phosphate (P_i_), 0.1-unit XOD, 0.5-unit PNP and 50-mM Tris-HCl, pH 7.4. For the radiometric assay of the hydrolase activity of NT5C2, incubations were performed in a medium containing 5-mM ATP, 20-mM MgCl_2_, 2-mM [8-^14^C]-IMP (5000 dpm/nmol), 100-mM Tris-HCl, pH 7.4 and 1.5-mM inosine in a total volume of 50 µL. At 0, 10, 20 and 30 min, the reactions were stopped by rapidly drying 10 µL of incubation mixture on PEI-cellulose precoated thin-layer plastic sheets; then, the chromatogram was developed in water to separate inosine from IMP. In this separation, the inosine standard was used and detected as ultraviolet adsorbing areas, which were excised and counted for radioactivity with 4-mL Optiphase Hisafe 2 scintillation liquid [26]. The phosphotransferase activity of NT5C2 was measured with a radioenzymatic assay [27]. The reaction mixture contained, in a total volume of 50 µL, 1.5-mM [8-^14^C]-inosine (3500 dpm/nmol), 2-mM IMP, 20-mM MgCl_2_, 5-mM ATP and 50-mM Tris-HCl, pH 7.4. The reaction was stopped at 0, 10, 20 and 30 min by spotting 10 µL of the incubation medium on DE-81 paper disks, which were washed once for 15 min in 1-mM ammonium formate and twice for 10 min in deionized water. The disks were dried and placed in counting vials filled with 4-mL Optiphase Hisafe 2 scintillation liquid.

One enzyme unit is the amount of enzyme that catalyzes the conversion of 1 µmol of substrate/min in the described conditions.

### 2.4. Measurement of Kinetic Parameters of Recombinant NT5C2

GraphPad Prism 8 (GraphPad Software, San Diego, CA, USA) was used to estimate the kinetic parameters, using a hyperbolic nonlinear regression analysis. The phosphotransferase method was used for the determination of K_M_ for inosine, K_50_ (concentration required to attain 50% of the effect) for ATP, K_50_ for Mg^2+^ and K_i_ for P_i_. The reaction mixtures contained 10 ng of the enzyme (recombinant WT or C547A mutant) and 0.2–3-mM [8-^14^C]-inosine, 1–9-mM ATP, 0.5–10-mM MgCl_2_ or 0.5–10-mM P_i_, respectively. The radioenzymatic hydrolase assay was used for the determination of K_M_ for IMP (10 ng of the enzyme) in the presence of 10–800-µM [8-^14^C]-IMP and for the measurement of k_cat_ (turnover number).

### 2.5. Measurement of Recombinant NT5C2 Activity at Different Adenylate Energy Charge Values

Phosphotransferase activity of NT5C2 was measured with and without 5-mM P_i_ in the presence of a mixture of various adenylic nucleotides (ATP, ADP and AMP) to give an adenylate energy value ranging from 0 to 0.9. The relative amounts of ATP, ADP and AMP (total final concentration 4 mM) at different energy charge values ([ATP] + 0.5 [ADP])/([ATP] + [ADP] + [AMP]) were calculated with the equilibrium constant of the adenylate kinase reaction equal to 0.8 [28].

### 2.6. Inactivation of NT5C2 in ADF Cells by H_2_O_2_

ADF cells were cultured on plates of 10-cm diameters with 10 mL of RPMI medium, supplemented with 10% FBS (*v/v*), 2-mM glutamine and 50-mU/mL penicillin/streptomycin. They were grown at 37 °C in a humidified atmosphere in the presence of 5% CO_2_. Approximately 9 million ADF cells (125,000/cm^2^) were transferred to 7 mL of Hank’s balanced salt solution and subjected to oxidative stress through three subsequent additions of 200-μM H_2_O_2_ every 30 min. After 4 h from the last addition of H_2_O_2_, the medium was removed, and the cells were washed with phosphate-buffered saline (PBS) to remove detached cells. The adherent cells were scraped, collected and lysed by three freeze/thaw cycles. The suspension was centrifuged at 10,000 *g* at 4 °C for 30 min, and the supernatant was referred to as the cell lysate. The phosphotransferase activity was assayed with 20 ng of cell lysate of both control and H_2_O_2_-treated cells. The assay was performed both in the absence and in the presence of 5-mM DTT.

### 2.7. Inactivation of Recombinant NT5C2 by CuCl_2_

The recombinant WT and mutant C547A NT5C2, at a final concentration of 0.08 mg/mL in 50-mM Tris-HCl, pH 7.4, were incubated both in the presence and absence of 300-µM CuCl_2_ for 15 min. An aliquot of the mixtures was withdrawn, and the hydrolase activity of NT5C2 was measured with the spectrophotometric assay. Five millimeters of DTT was added to the mixtures treated with CuCl_2_, and after 15 min, an aliquot was withdrawn, and the hydrolase activity was measured.

### 2.8. NT5C2/IPAF Proximity Ligation Assay in 2-dG-Exposed A549 Cells

A549 cells were cultured in DMEM high glucose (25 mM) supplemented with 10% FBS, 1% glutamine, 100 U/mL penicillin and 100 U/mL streptomycin. They were grown at 37 °C in a humidified atmosphere in the presence of 5% CO_2_. For the NT5C2/IPAF PLA, A549 cells were plated (10,000 per well) on a Nunc™ Lab-Tek™ ChamberSlide System, allowed to adhere for the next 12 h and subsequently exposed for 48 h to increasing concentrations of 2-dG (0.08–10 mM). NT5C2/IPAF interaction was assayed with PLA, as previously reported [24]. Cells were washed twice with PBS, fixed (4% paraformaldehyde for 20 min), permeabilized (0.1% Triton for 30 min), blocked (1% FBS plus 0.1% bovine serum albumin for 30 min) and incubated with primary antibodies against NT5C2 and IPAF at 4 °C overnight. The day after, cells were incubated with the appropriate DNA-linked secondary antibody, and in-situ PCR amplification was performed using the PLA technology according to the manufacturer’s instructions. The in-situ PLA signal was analyzed by confocal microscopy. The images were acquired by a Laser Scanning Confocal Microscope (TCS-NT Leica Microsystems, Wetzlar, Germany). The fluorescently labeled oligonucleotides incorporated in the Rolling Circle Amplification (RCA) step of PLA were detected using for excitation the 543 ± 10-nm wavelength line of the argon-ion laser combined with a narrow bandpass emission filter at 610 ± 10 nm. The laser power was kept at 10% of the maximal power to avoid the photo-bleaching of the fluorescent probes. Images were acquired at 1024 × 1024-pixel resolution with a 63× (1.4 NA) oil immersion objective and averaged 4 times to improve the signal/noise ratio. For each field, both fluorescent and transmitted light images were acquired on separate photomultipliers. As the negative control, the same protocol was applied to cells exposed to one primary antibody only or none of them.

### 2.9. Preparation of A549 Cell Lysates for ELISA Test and HPLC Analysis

A549 cells were seeded at a density of 3.3 × 10^4^ cells/cm^2^ in 10-mL RPMI medium in 100-mm-diameter plates and allowed to adhere for the next 12 h. Subsequently, cells were exposed to 0.08-, 0.2-, 2- and 10-mM 2-dG and incubated for 48 h at 37 °C in a humidified atmosphere in the presence of 5% CO_2_. For the quantification of NT5C2 and IPAF by ELISA test, cells were washed with PBS, trypsinized and resuspended in 300-µL 100-mM Tris-HCl, pH 7.4 in the presence of protease inhibitors. Cell lysates were obtained by three freeze/thaw cycles followed by centrifugation at 10,000× *g* for 40 min at 4 °C. For the quantification of intracellular adenylic compounds by HPLC, cells exposed to 2-dG were treated as previously described for the extraction of nucleotides [29]. The analysis was performed by HPLC according to Micheli et al. [30]. Intracellular energy charge (EC) was calculated using the following formula: ([ATP] + 1/2 [ADP])/([ATP] + [ADP] + [AMP]).

### 2.10. ELISA Test

ELISA test was performed on cell lysates obtained from both ADF cells (in the absence and in the presence of 200-µM H_2_O_2_) and A549 cells (treated with different amounts of 2-dG). Cell lysates were placed in a 96 multi-well plate and kept at 4 °C overnight. For the quantification of NT5C2, 30 µg of ADF and 10 µg of A549 cell lysate were used. For the quantification of IPAF, 40 µg of A549 cell lysate was used. Lysates were washed three times with PBS + 0.1% Tween-20 (Solution A), incubated with blocking solution (5% dry nonfat milk in PBS + 0.1% Tween 20) for 2 h with gentle shaking, washed three times with solution A, incubated with antibody against NT5C2 (1:1000) or against IPAF (1:100) and kept overnight at 4 °C with gentle shaking. Successively, they were washed three times with solution A, incubated with HRP-linked secondary antibodies (1:2000 for NT5C2 and 1:5000 for IPAF) for one hour with gently shaking, washed three times with Solution A and incubated with 100 µL of developing solution TMB for 10 min, and then 100 µL of STOP solution was added. The absorbance was measured at 405 nm using EL 808 Ultramicroplate Reader (Bio-Tek Inc., Colmar, France)

### 2.11. Other Methods

The protein content was determined following the method described by Bradford [31]. All data are reported as the mean ± SD. Significant differences among groups were determined using two-way analysis of variance (ANOVA) followed by Tukey’s multiple comparison test with GraphPad Prism 8. A *p*-value < 0.05 was considered to indicate statistical significance.

## 3. Results

### 3.1. The NT5C2 Activity Is Dependent on Adenylate Energy Charge

**Figure 1:**
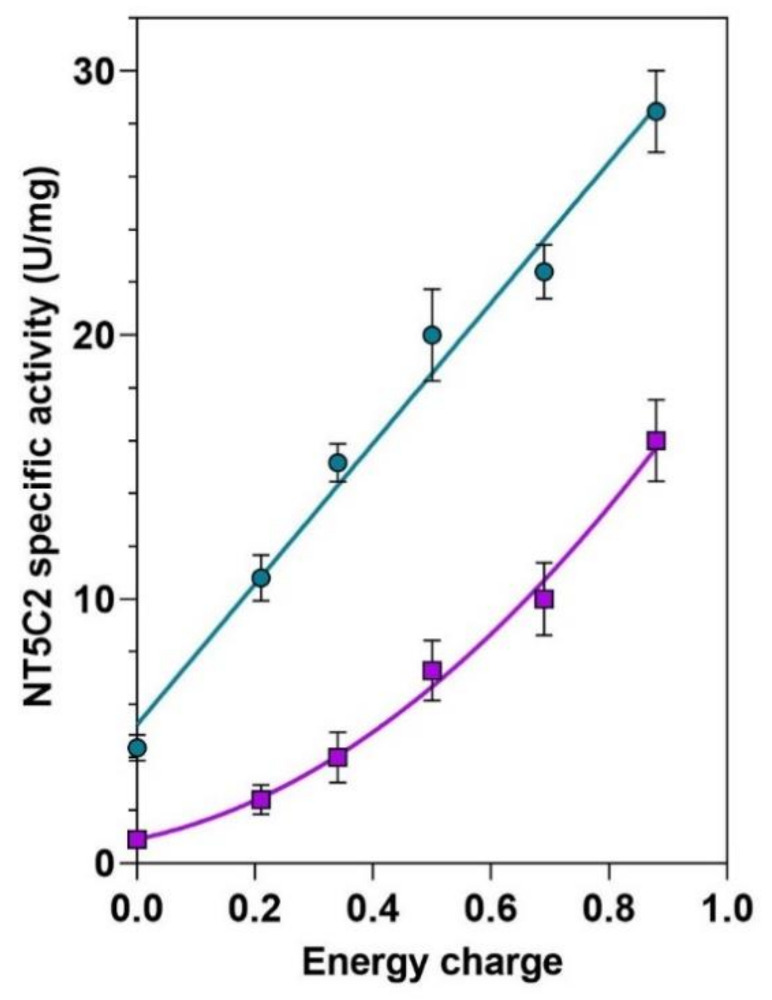
NT5C2 activity is dependent on the adenylate energy charge. The phosphotransferase activity of wild-type (WT) recombinant NT5C2 was evaluated in vitro at different energy charge values obtained with various concentrations of ATP, ADP and AMP, both in the absence (○) and in the presence (□) of 5-mM Pi. Results are the mean + SD of three independent experiments.

The recombinant WT bovine NT5C2, which exhibits a 99% homology with the human enzyme [24], was assayed in the presence of a mixture of ATP, ADP and AMP at different concentrations simulating different energy charges. Figure 1 shows that NT5C2 activity is strictly dependent on the adenylate energy charge, increasing approximately 6 and 16-fold in the absence and presence of Pi, respectively, when the adenylate energy charge raises from 0 to 0.9. In the presence of physiological P_i_ concentrations [32], the sigmoidicity of the curve is emphasized, probably because the inhibitory effect of phosphate is more accentuated at a low energy charge, where the low concentration of ATP cannot overcome the P_i_ inhibition [27].

### 3.2. Effect of Oxidative Stress on NT5C2 in ADF Cells

**Figure 2:**
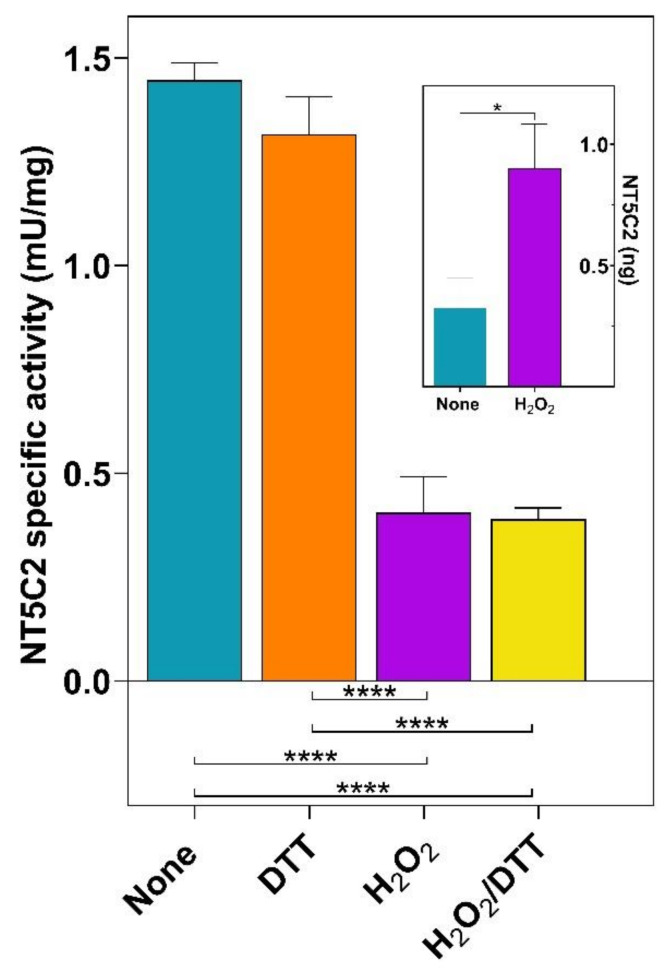
Effect of oxidative stress on the activity of NT5C2 in ADF cells. ADF cells were subjected to oxidative stress by incubation with 200-μM H_2_O_2_. Both the control (none) and treated (H_2_O_2_) cell lysates, obtained as described in “Methods”, were assayed for the phosphotransferase activity of NT5C2 in the absence and in the presence of 5-mM DTT. The actual amount of NT5C2 present in the lysates obtained from the control (none) and treated (H_2_O_2_) ADF cells was evaluated by the ELISA test (inset). Results are the mean + SD of at least three independent experiments. Statistical significance: **** *p* ˂ 0.0001 and * *p* ˂ 0.05.

In order to adapt to an altered redox status, cancer cells often exhibit an increased antioxidant capacity [33]. We used an astrocytoma cell line, ADF, whose capacity to counteract oxidative stress conditions has been previously reported [34], to evaluate whether the NT5C2 activity is susceptible to oxidative stress conditions. The ADF cells were subjected to H_2_O_2_ treatment, and the phosphotransferase activity of NT5C2 was measured in the cell lysates. The oxidative conditions caused a significant loss of enzyme activity (1.45 ± 0.04 mU/mg vs. 0.41 ± 0.09 mU/mg), and the effect was not reversed by the addition of DTT in the assay mixture (Figure 2). Despite the loss of activity, the ELISA test demonstrated that the concentration of the enzyme increased in cells exposed to H_2_O_2_ (Figure 2, inset), indicating that the decrease of activity is not related to a loss of the protein but, rather, to the protein modification.

Since, in a previous paper, an involvement of a disulphide bridge between C175 and C547 in NT5C2 inactivation was hypothesized [7], a mutated recombinant enzyme was obtained by substituting cysteine 547 with alanine. The C547A mutated enzyme was purified to electrophoretic homogeneity with a higher yield in terms of protein than that obtained for the WT enzyme (0.27 ± 0.12 mg/mL WT vs. 1.05 ± 0.28 mg/mL C547A; *p* ˂ 0.001). The kinetic parameters: K_M_ for inosine and IMP, K_50_ for the activators ATP and Mg^2+^ and K_i_ for the negative effector P_i_, were comparable, while the mutated enzyme exhibited a turnover number approximately five-fold higher than the WT enzyme (Table 1).

**Figure 3:**
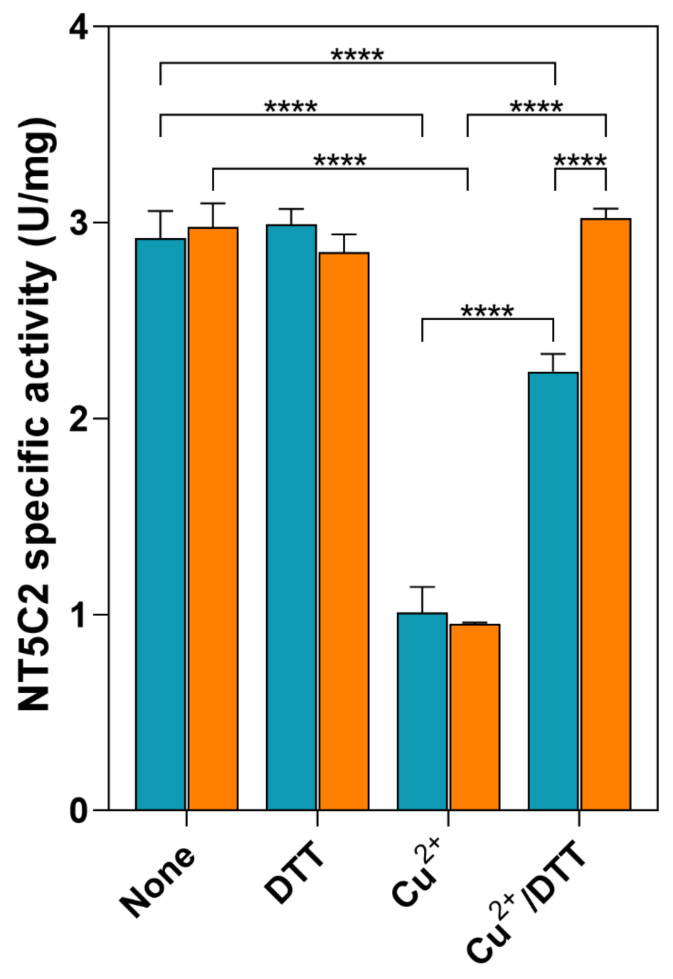
Effect of oxidative stress on the activity of recombinant WT and C547A NT5C2. The activity of recombinant NT5C2, both the WT (blue bars) and C547A mutant (orange bars), was measured after incubation in the absence (none) and the presence of either 5-mM dithiothreitol (DTT) or of 300-µM CuCl_2_ (Cu^2+^)_._ The activity was also measured after the addition of 5-mM DTT to the mixtures treated with 300-µM CuCl_2_ (Cu^2+^/DTT). Results are the mean ± SD of at least three independent experiments. Statistical significance: **** *p* ˂ 0.0001.

We hypothesized that these results might be due to both a higher solubility of the mutated enzyme and its impossibility to form the inactivating disulphide bridge. Upon storage, the recombinant C547A enzyme loses its activity at a faster rate than the WT NT5C2. Therefore, the measured specific activity of the enzyme is dependent on the time elapsed from purification. To test whether the oxidative conditions brought about an inactivation of NT5C2 through the formation of the above-mentioned disulphide bridge, the recombinant enzymes were incubated in the presence of 300-µM CuCl_2_ for 15 min, and the activity was measured both in the absence and in the presence of 5-mM DTT (Figure 3). Both the WT and C547A enzymes decrease their activity to the same extent upon incubation with Cu^2+^, and, in the presence of DTT, the mutant recovered all the activity, while the WT recovered approximately 70% of the activity (Figure 3). This demonstrated that the oxidative conditions exert multiple effects on the enzyme, in which the disulphide bridge plays only a small role.

### 3.3. Interaction of NT5C2 with IPAF in A549 Cells Is Affected by Oxidative Stress and Energy Charge

**Figure 4:**
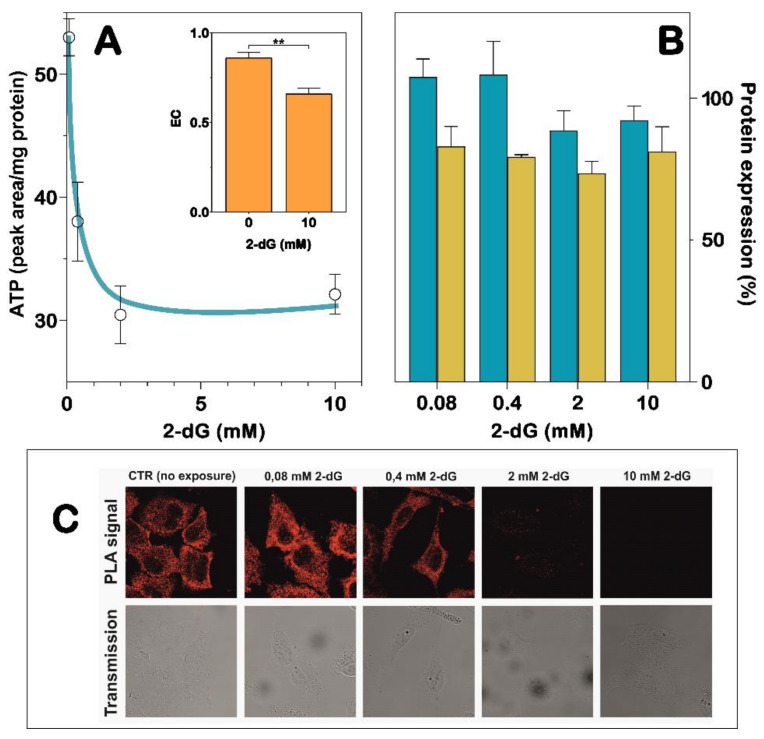
Effect of oxidative stress conditions on the NT5C2/IPAF interaction. A549 cells were incubated in the presence of increasing concentrations of 2-dG (0.08–10 mM) for 48 h. (**A**) Cells exposed to 2-dG were submitted to an extraction of nucleotides, and the ATP content was measured by HPLC analysis. The inset shows the adenylate energy charge (EC) values estimated in cells exposed to 0- and 10-mM 2-dG. Results are the mean ± SD of three independent experiments and are expressed as the peak area normalized in the protein content. Statistical significance: ** *p* < 0.001. (**B**) ELISA test was performed on A549 cell lysates obtained from cells incubated with 2-dG. The protein content is reported as a percentage of the control: NT5C2 (blue bars) and IPAF (yellow bars). Results are the mean ± SD of three independent experiments. (**C**) Representative confocal images of the in-situ proximity ligation assay (PLA) in A549 cells. Upper lane: PLA allows the direct visualization and the relative quantification of the NT5C2/IPAF interaction within single cells. The fluorescent red dots indicate the sites of close proximity and the molecular interaction between NT5C2 and IPAF. Lower lane: non-confocal bright field images of the same fields shown in the upper lane.

We previously demonstrated by PLA and coimmunoprecipitation that NT5C2 can interact with IPAF in A549 cells [24]. Recently, it was also observed that the incubation of A549 cells with 2-dG, a good inhibitor of both glycolysis and the pentose phosphate pathway [35], causes oxidative stress by lowering the energy charge and increasing the mitochondrial oxidative stress [8]. Additionally, the NT5C2 activity decreased as a consequence of oxidative stress, which determined an irreversible inactivation of the enzyme [8]. Therefore, we incubated A549 cells in the presence of increasing concentrations of 2-dG, a condition that stabilizes a less active enzyme conformation [8]. Figure 4A shows that, as expected, the incubation with increasing concentrations of 2-dG causes a decrease of the ATP content in A549 cells. Although both IPAF and NT5C2 are expressed in A549 cells, and the presence of 2-dG, at a concentration ranging from 0.08 to 10 mM, does not cause a decrease of both proteins (Figure 4B), NT5C2 and IPAF lose the ability to interact in a 2-dG concentration-dependent manner (Figure 4C). This indicates that the conformational change of NT5C2, which occurs as a consequence of the change in the energy charge and oxidative stress, prevents protein–protein interactions.

## 4. Discussion

Cell or animal models in which a protein is silenced or overexpressed may represent a valuable tool to evaluate the consequences of mutations with a loss or gain of function at the cellular level, but the use of these techniques to study the physiological functions of that specific protein is questionable. In fact, the overexpression of NT5C2, our protein of interest, may have different effects, depending on the final specific activity attained by the enzyme in the cellular model. A moderate increase of NT5C2 activity (two-five-fold) in ADF cells causes an increase of cell proliferation [16], while a strong increase of enzyme activity in acute lymphoblastic leukemia cells leads to nucleotide degradation and to an inhibition of proliferation [36]. Therefore, our interest was focused on finding the molecular consequences of enzyme regulation possibly related to its conformational changes [4]. We relied on cultured cell models in which more- and less-active NT5C2 conformations were stabilized by high-energy charges and oxidative stress, respectively. Firstly, we demonstrated that the recombinant purified NT5C2, in the presence of physiological concentrations of the inhibitor P_i_, is very sensitive to changes in the energy charge, especially above 0.4, where the steepness of the sigmoidal curve is the maximal. To demonstrate the dependence of NT5C2 activity on the oxidative state of the cell, we used an astrocytoma cell line, ADF, which, like several cancer cells, is well-equipped to counteract oxidative stress [34]. ADF cells were incubated with hydrogen peroxide following a protocol previously described to study the sensitivity to the oxidative stress of several enzymes involved in the detoxification of reactive oxygen species (ROS) in the same cell line [34]. In a previous paper, using the same conditions described in the present investigation, several enzymes, such as NADPH-dependent reductases, measured with different substrates have been reported to be insensitive to oxidative treatment, and NAD-dependent dehydrogenases were easily rescued by the addition of DTT [34]. Conversely, the NT5C2 activity in ADF cells incubated with hydrogen peroxide decreased approximately 70% and could not be rescued by incubation with DTT, thus confirming the extreme sensitivity of NT5C2 to oxidative conditions. The ELISA analysis demonstrated that, despite the loss of NT5C2 activity, the expression of the enzyme protein increased in cells exposed to hydrogen peroxide, possibly reflecting an attempt of the cells to counteract the inactivating effect of hydrogen peroxide on the NT5C2 activity. Indeed, we previously demonstrated that, in ADF cells, a transitory silencing of approximately 50% of NT5C2 induced apoptotic cell death within 72 h, thus indicating the relevance of this enzyme for the cell life [37]. This is, however, cell- and/or tissue-specific, as several other models (cells and animals) have been reported to remain viable, even with the partial or complete depletion of NT5C2 [12,13,14,15,16,38].

Previous results suggested that the formation of a disulphide bridge (C175 and C547) might be responsible for NT5C2 inactivation [7]. To test the hypothesis of the possible involvement of this disulphide bridge in the reversible oxidation of NT5C2, we prepared a mutant (C547A) incapable of forming the disulphide bridge and incubated both the mutant and WT recombinant NT5C2 with CuCl_2_. Indeed, several experimental lines of evidence indicate the occurrence of protein oxidation as a consequence of copper action [39,40]. The results indicated that both mutant and WT enzymes were equally sensitive to the pro-oxidant conditions and lost a comparable amount of activity, but the failure of the mutant enzyme to form the disulphide bridge improved the ability of DTT to rescue the mutant inactivated enzyme. In our opinion, the disulphide bridge stabilizes a conformation that increases the NT5C2 sensitivity to pro-oxidant conditions. To further investigate the effect of the energy charge and oxidant conditions upon NT5C2 conformation, we used a human lung carcinoma cell line (A549) expressing the WT NT5C2 enzyme. In a previous paper, we showed that A549 cells incubated in the presence of 2-dG exhibit signs of oxidative stress, including a loss of NT5C2 activity, while maintaining the protein expression at the same level of the control cells [8]. In particular, these cells showed a significant decrease in glutathione content and an increase in mitochondrial reactive oxygen species, both indicative of oxidative stress by 2-dG [8]. When A549 cells were incubated in the presence of an increasing concentration of 2-dG, a concentration-dependent loss of intracellular ATP with a concomitant decrease in the energy charge was observed. This is not surprising, since 2-dG is a well-known inhibitor of both glycolysis and the pentose phosphate pathway [35]. In these experimental conditions, NT5C2 was stabilized in a conformation compatible with a low-energy charge and high oxidative aggression and was unable to interact with IPAF. Conversely, as reported in a previous paper [24] and confirmed in this paper, an interaction of NT5C2 with IPAF was clearly demonstrated in A549 cells grown in the absence of 2-dG. Therefore, we might hypothesize that, at a high-energy charge, NT5C2 can contribute to keep the inflammatory response below alarming levels by preventing IPAF oligomerization through interaction with the LRR domain [24]. Conversely, at a low-energy charge, NT5C2 leaves the target protein free to activate the inflammatory response, if necessary. As mentioned above, more than 40 possible interactors for NT5C2 can be found in the databases (BioGRID and IntAct-EMBL-EBI). Since the structure of the enzyme depends on the energy charge, as well as on the reducing environment, we may hypothesize that NT5C2 acts as a sensor of the health of the cell. Indeed, the change of conformation of NT5C2 affects its ability to bind the target protein(s), and this may reflect on a different intracellular signaling, which might contribute to the metabolic adjustments of the cell.

## Figures and Tables

**Table 1:** Kinetic parameters of the wild-type (WT) and C547A recombinant NT5C2.

Kinetic Parameters	WT	C547A
K_M_ Ino (mM)	1.00 ± 0.059	0.83 ± 0.061
K_M_ IMP (mM)	0.10 ± 0.018	0.12 ± 0.022
K_50_ Mg^2+^ (mM)	2.00 ± 0.114	0.60 ± 0.101
K_50_ ATP (mM)	2.00 ± 0.190	1.00 ± 0.087
K_i_ P_i_ (mM)	2.00 ± 0.156	2.00 ± 0.218
k_cat_ (sec^−1^)	52.90 ± 3.35	228.80 ± 16.56

Results are the mean ± SD of three independent experiments. Ino: inosine.

## Data Availability

The data that support the findings of this study are available from the corresponding author upon reasonable request.
